# Microcrystallography, high-pressure cryocooling and BioSAXS at MacCHESS

**DOI:** 10.1107/S0909049510036010

**Published:** 2010-11-05

**Authors:** Ulrich Englich, Irina A. Kriksunov, Richard A. Cerione, Michael J. Cook, Richard Gillilan, Sol M. Gruner, Qingqui Huang, Chae Un Kim, William Miller, Soren Nielsen, David Schuller, Scott Smith, Doletha M. E. Szebenyi

**Affiliations:** a MacCHESS (Macromolecular Diffraction Facility at CHESS), Cornell University, Ithaca, NY 14853, USA; b Department of Chemistry and Chemical Biology, Cornell University, Ithaca, NY 14853, USA; c Cornell High Energy Synchrotron Source (CHESS), Cornell University, Ithaca, NY 14853, USA; d Field of Biophysics, Cornell University, Ithaca, NY 14853, USA; e Physics Department, Cornell University, Ithaca, NY 14853, USA

**Keywords:** protein crystallography, microcrystallography, BioSAXS, cryo, pressure

## Abstract

The Macromolecular Diffraction Facility at the Cornell High Energy Synchrotron Source (MacCHESS) is a national research resource supported by the National Center for Research Resources of the US National Institutes of Health. MacCHESS is pursuing several research initiatives designed to benefit both CHESS users and the wider structural biology community. Three initiatives are presented in further detail: microcrystallography, which aims to improve the collection of diffraction data from crystals a few micrometers across, or small well diffracting regions of inhomogeneous crystals, so as to obtain high-resolution structures; pressure cryocooling, which can stabilize transient structures and reduce lattice damage during the cooling process; and BioSAXS (small-angle X-ray scattering on biological solutions), which can extract molecular shape and other structural information from macromolecules in solution.

## Abstract 2

Three research initiatives pursued by the Macromolecular Diffraction Facility at the Cornell High Energy Synchrotron Source (MacCHESS) are presented.

## 1. Introduction

The X-ray solution of macromolecular structures involves several steps. If the macromolecule can be crystallized, then crystals of suitable size and diffraction quality must be obtained. In general these must then be cryoprotected to mitigate radiation damage. If crystals cannot be obtained, then low-resolution structural information may still be available by BioSAXS. BioSAXS can also help phase crystalline diffraction patterns. Below, we describe technical developments at CHESS in each of the three areas microcrystallography, cryocooling and BioSAXS.

## 2. Microcrystallography

It is now very common for users to arrive at CHESS with crystals that are either small or have very small diffracting regions. Crystals 50 µm across are now routine and often crystals are only 10 to 20 µm in some dimension. In addition, for large inhomogeneous crystals, better diffraction is sometimes obtained by illuminating a small well ordered portion rather than the whole crystal. Small-area illumination also allows minimization of the effects of radiation damage by moving to fresh portions of the crystal when the diffraction deteriorates.

**Figure 1:**
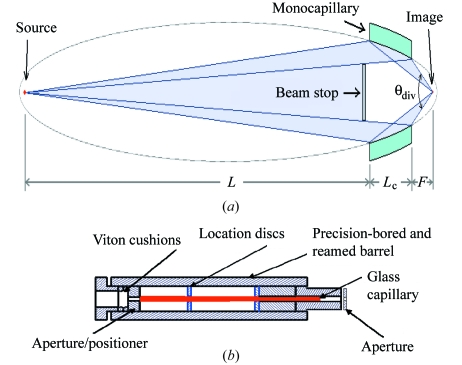
(*a*) Diagram of a cross section of a microfocusing glass capillary; the capillary is drawn to an elliptical internal profile. A divergent X-ray source located in the left focus of the ellipse is reflected on the inside of the glass capillary (shown here in green) towards the second focus of the ellipse, where the sample resides (*L* is the distance from the source, *L*_c_ the actual length of the capillary, *F* the focal length; the angle θ_div_ describes the beam divergence at the sample). A beamstop centered upstream of the capillary blocks the direct beam. (Diagram courtesy of S. Cornaby, PhD thesis 2008, Cornell University, USA.) (*b*) A glass capillary (red) mounted in a precision-bored and reamed brass barrel. The capillary is guided in the front and back in two brass bores while being supported in the center by two Viton O-rings (blue) at 1/4 and 3/4 of its length. A brass cap in the back pushes onto a Viton cushion that holds the capillary in place. A clean-up aperture made of lead is added onto the front of the brass barrel.

Kirkpatrick–Baez (KB) mirrors are most commonly used to produce synchrotron microbeams. KB mirrors are less practical at CHESS because of space limitations and a source size that is large and relatively divergent. In consequence, CHESS has developed single-bounce focusing monocapillary optics to generate intense microbeams (Bilderback *et al.*, 2007 ▶; Gillilan *et al.*, 2010 ▶). Capillaries are drawn to order on an in-house computer-controlled drawing tower. The drawing tower apparatus can be programmed to produce capillaries of a desired focal spot size and working distance to match beamline characteristics and crystal requirements. MacCHESS routinely uses 20 µm and 5 µm capillaries with modest beam divergences of 2–4 mrad, which allow collection of data on samples with lattice parameters up to about 500 Å. For example, a typical total flux of 2.8 × 10^10^ photons s^−1^ for a 20 µm capillary has been measured at the CHESS F1 station (Gillilan *et al.*, 2010 ▶), while the weaker bending-magnet station F3 equipped with a 5 µm capillary produces 3.2 × 10^9^ photons s^−1^ (Englich, 2008 ▶). The capillaries are mounted in a housing that allows for easy exchange between collimator and microfocusing capillary [Figs. 1(*a*) and 1(*b*) ▶].

**Figure 2:**
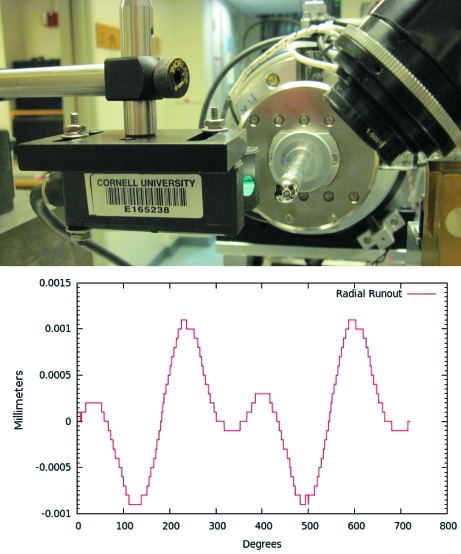
(Top) A Keyence laser device measures the runout of the air-bearing rotation stage as it reflects from a calibrated polished steel sphere (Bal-tek, Los Angeles, CA; http://www.precisionballs.com/). (Bottom) Radial runout of the stepper stage with encoders. The runout determined by this set-up over two full revolutions is ±1 m.

Efficient data collection from microcrystal regions also requires advanced positioning and visualization tools. Precise positioning is necessary to keep a microcrystal centered in the X-ray beam as it rotates. The MacCHESS staff have improved the performance of the air-bearing-based rotation stage. To measure the sphere of confusion, a new Keyence laser device replaces a mechanical probe and allows for fast and accurate automated data acquisition (Fig. 2 ▶). A runout of ±1 µm has been achieved by applying tight mechanical standards in conjunction with encoded stepper motors. Equally important is the implementation of a high-resolution crystal-viewing camera system with an accompanying Java-based computer interface for manual and automated crystal centering.

## 3. High-pressure cryocooling

Flash-cooling of biological macromolecules has been the method of choice to reduce radiation damage and prolong the lifetime of the sample in intense synchrotron beams (Hope, 1988 ▶). The method typically involves soaking the crystals in a cryoprotectant solution prior to cryocooling. Determining the right kind and amount of cryoprotectant can be a tedious task. An alternative is a novel high-pressure cyrocooling procedure that requires no, or very little, cryoprotectant (Kim *et al.*, 2005 ▶). The method involves cooling macromolecular crystals to cryogenic temperatures (∼100 K) in high-pressure (up to 200 MPa) helium gas. Applications include successful cryocooling with little or no penetrating cryoprotectant, and native sulfur SAD (single-wavelength anomalous dispersion) phasing. Samples in capillaries can also be pressure cryocooled (Kim *et al.*, 2007 ▶; Chen *et al.*, 2009 ▶). The method has been extended to other gases, *e.g.* Kr or Xe (followed by He) for SAD phasing (Kim *et al.*, 2006 ▶, 2007 ▶), and CO_2_ (alone at lower pressure) to visualize an enzymatic intermediate state in carbonic anhydrase (Domsic *et al.*, 2008 ▶). Surprising results include visualization of ligands which could not be seen using conventional flash-cooling methods (Albright *et al.*, 2006 ▶), and unusual phase behavior of water in protein crystals (Kim *et al.*, 2008 ▶, 2009 ▶). The method can also be used to study pressure effects on protein structures (Barstow *et al.*, 2008 ▶, 2009 ▶). A mechanism involving high-density amorphous ice has been proposed to explain why the method works (Kim *et al.*, 2005 ▶, 2008 ▶, 2009 ▶).

**Figure 3:**
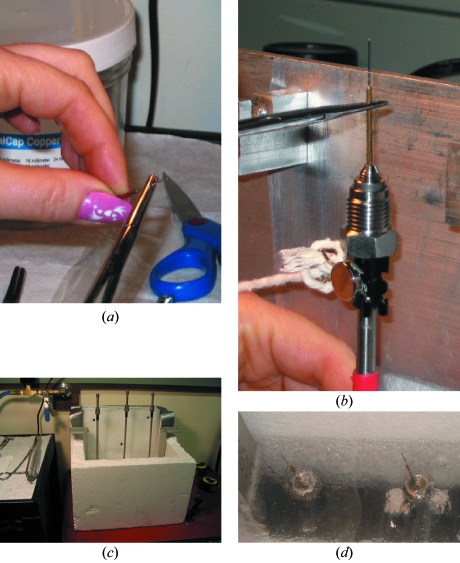
Steps in manipulation of a crystal for pressure cryocooling. (*a*) The crystal is mounted in a standard cryoloop on a steel pin and a piano wire is attached. (*b*) The pin with crystal is placed into a steel pressure tube. A magnet on the outside holds the pin in place. The pressure tubes are connected to the manifold and lowered into the liquid-nitrogen bath [panel (*c*) shows tubes and bath, with manifold removed for clarity]. (*d*) After pressure cooling the samples are removed from the pressure tubes under liquid nitrogen.

The procedure, as described earlier (Kim *et al.*, 2005 ▶), requires samples to be coated with a thin film of hydrocarbon oil for protection against drying, pressurized in a helium atmosphere, and then flash-cooled to 77 K. Details are described in the following steps [Figs. 3(*a*)–3(*d*) ▶]:

(i) Mount sample (usually a crystal in oil) in a cryoloop on a steel pin (Fig. 3*a* ▶) with a piece of piano wire attached to the base.

(ii) Slide the pin with the mounted sample into pressure tubing (Fig. 3*b* ▶), where an external magnet holds it near the top.

(iii) Place up to three tubes into a bath partially filled with liquid nitrogen (Fig. 3*c* ▶).

(iv) Place the bath in a safety enclosure.

(v) Connect tubes to a manifold and pressurize the system with helium, usually to about 200 MPa (2 kbar).

(vi) Remove magnets, letting the pins fall to the bottom of the tubes (at 77 K).

(vii) Release pressure from the system.

(viii) Disconnect the tubes at top and bottom, keeping the pins with the samples under liquid nitrogen (Fig. 3*d* ▶).

The pins can then be transferred under liquid nitrogen to standard bases and handled like conventional flash-cryocooled samples. Note that the procedure can be used to cryocool samples in capillaries within the mother liquor (Kim *et al.*, 2007 ▶).

**Figure 4:**
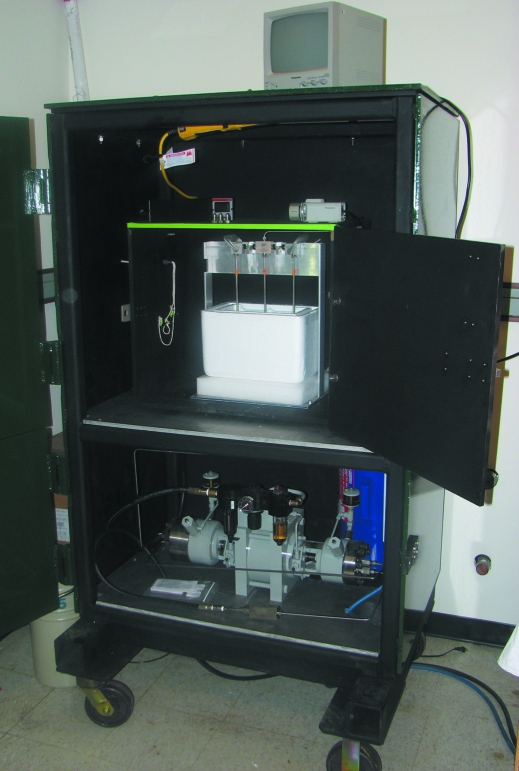
A large mobile steel cabinet (188 cm × 112 cm × 74 cm) houses the pressure cryocooling apparatus. A safety enclosure on the upper shelf contains the liquid-nitrogen bath and manifold for the steel tubes with the samples. The lower shelf carries the pump that generates a maximum pressure of 200 MPa.

The original high-pressure cryocooling apparatus is housed in the Cornell Physics Department. In order to make the high-pressure cryocooling method more available to users, a second apparatus, capable of operation to 200 MPa, has been made and installed at the synchrotron, and staff members have been trained in its use. All high-pressure components are enclosed in a steel cabinet with half-inch-thick walls designed to safely contain the apparatus in case of an unexpected gas release or failure of a high-pressure part. The whole apparatus weighs in at about 1500 kg (Fig. 4 ▶).

## 4. BioSAXS

**Figure 5:**
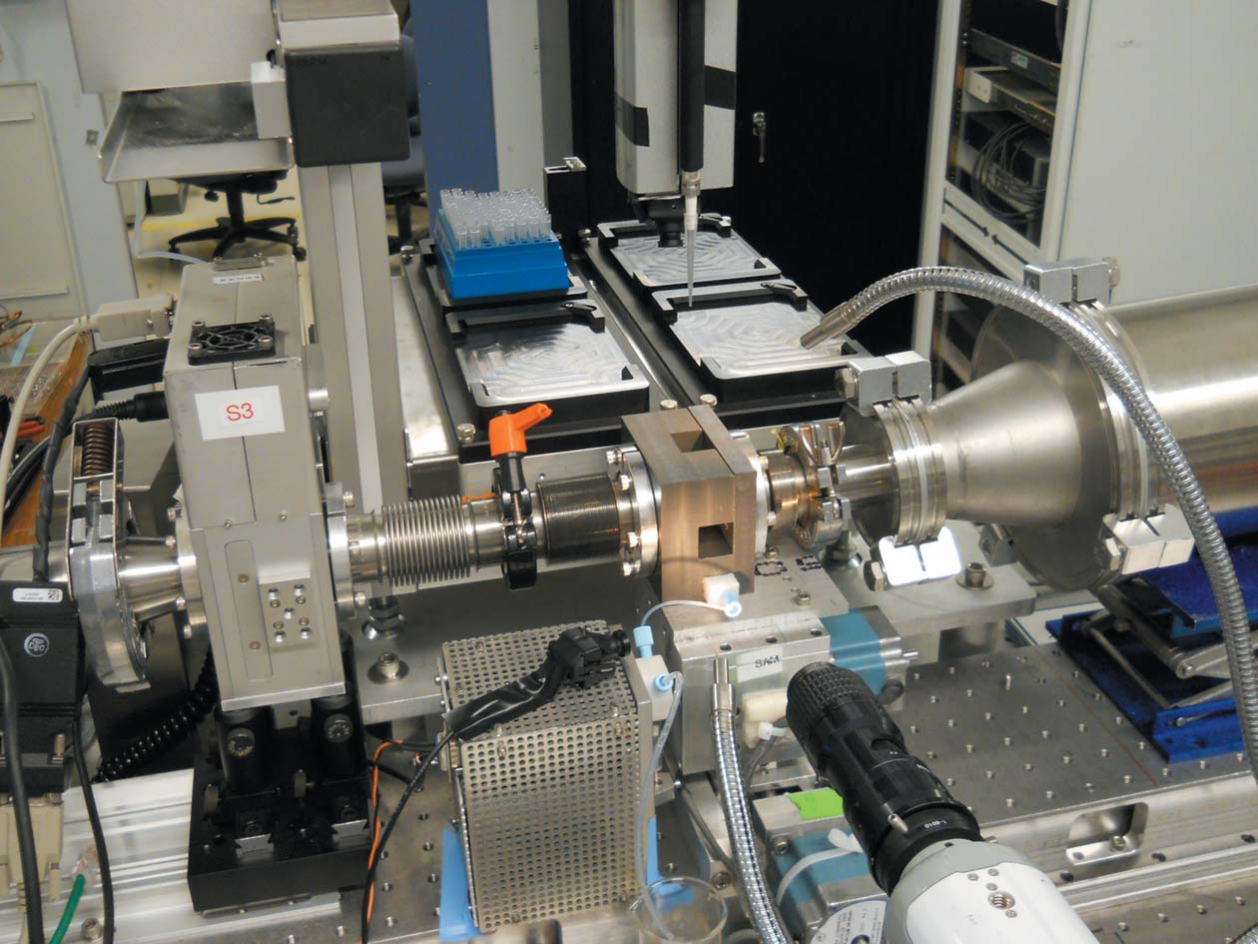
The BioSAXS set-up, shown here at CHESS G1 line, includes a quartz capillary flow cell housing (block at center) with a port at the top through which the robotic pipetting system (top) loads a sample. The X-ray beam travels entirely through vacuum from left to right in this figure. The video camera (front) provides a transverse *in vacuo* view of the flow cell for sample alignment.

**Figure 6:**
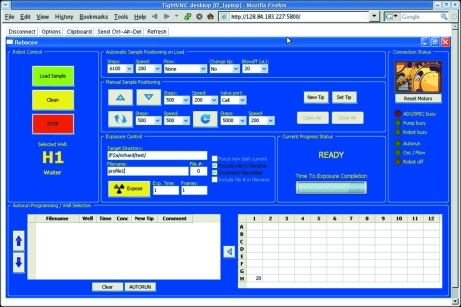
BioSAXS robot control software. The Python-based graphical user interface controls the pipetting action of the robot, the pumping which positions the sample in the beam, and the X-ray exposure. By using disposable pipette tips to load the flow cell, sample cross contamination and loss are minimized. The sample can be oscillated in the beam or flowed at a constant rate to reduce radiation damage.

Small-angle X-ray scattering on biological solutions (BioSAXS) is rapidly growing in user demand. It continues to play an increasingly important role in structural biology, not only as an aid in the assembly of macromolecular complexes but also in understanding changes in oligomeric states, determining conformation in solution and assessing structural integrity. Low-resolution shapes may be obtained for large macromolecules and macromolecular complexes without growing crystals. BioSAXS also allows studies of conformational changes of proteins and other macromolecules in solution under a very wide range of conditions including those which are close to physiological. In the past, BioSAXS has been supported at MacCHESS on a limited basis through access to the CHESS G1 line. A solid-state cooled housing using specially designed disposable sample cells (Ando *et al.*, 2008 ▶) has been used successfully in a number of cases (Gupta *et al.*, 2010 ▶; Navarro *et al.*, 2009 ▶; Bennett *et al.*, 2008 ▶). MacCHESS is currently commissioning a dedicated BioSAXS beamline at the CHESS F2 line equipped with high-flux multilayer optics, robotic sample loading and microfluidic sample handling. High-precision specially polished slits (Advanced Design Consulting USA) have been mounted upstream *in vacuo* to reduce parasitic scattering. A compact commercial pipetting robot (Hudson Robotics) has been adapted for automatic sample loading (Fig. 5 ▶). Users preload samples into standard 96-well plates and the robotic system delivers them to a specially designed entry port where they are aspirated into a vacuum-enclosed thin-walled quartz capillary for X-ray exposure. While sample volumes as low as 15 µl can be loaded automatically, volumes as low as 5 µl have been loaded into the system manually. By flowing the sample past the X-ray beam at a controlled rate, the effects of radiation damage are dramatically reduced. Open-source software based on the BioXTAS RAW project (Nielsen *et al.*, 2009 ▶) has been adapted to handle both robotic control and data processing (Fig. 6 ▶). To date, the robotic system has been tested on both the F2 and G1 beamlines, and is available to users. The major upgrade of the F2 beamline optics, which will significantly improve BioSAXS performance at that station, is planned for Spring 2011 with the addition of high-flux multilayer optics.

## 5. Summary

MacCHESS has pioneered variations on steps to obtain biomolecular structures at synchrotron sources. The use of ‘drop-in’ single-bounce monocapillary optics offers users great flexibility to choose a variety of beam sizes at a given beamline. Moreover, these optics are readily retrofit into existing beamlines with relatively little effort. High-pressure cryocooling allows users to obtain better data in many cases where diffraction quality is compromised by conventional cryocooling procedures. Solution SAXS capabilities at CHESS complement standard crystal diffraction capabilities. It is not unusual at CHESS for users to simultaneously perform crystal diffraction and solution SAXS experiments on a given protein, on appropriately equipped beamlines, at the same time.
